# Concentrations of Selected Adipocytokines in the Blood Plasma in Proximal Suspensory Desmopathy of Horses, with a Focus on Their Physical Activity—A Pilot Study

**DOI:** 10.3390/ijms25010205

**Published:** 2023-12-22

**Authors:** Beata Nowicka, Anna Torres, Izabela Polkowska, Jagoda Jackow-Nowicka, Maciej Przewozny, Joanna Jackow-Malinowska

**Affiliations:** 1 Department and Clinic of Animal Surgery, University of Life Sciences in Lublin, Głeboka 30, 20-612 Lublin, Poland; iza-polkowska@tlen.pl; 2 Department of Pediatric and Adolescent Gynecology, Medical University of Lublin, Chodzki 4, 20-094 Lublin, Poland; annatorres@umlub.pl; 3 Department of General and Interventional Radiology and Neuroradiology, Wroclaw Medical University, ul. Borowska 213, 50-556 Wrocław, Poland; 4 Equi Vet Serwis, Wygoda 6, 64-320 Buk, Poland; m.przewozny@klinika-konie.com.pl; 5 St John’s Institute of Dermatology, King’s College London, London SE1 9RT, UK; joanna.jackow@kcl.ac.uk

**Keywords:** proximal suspensory desmopathy, adipokines, Achilles

## Abstract

Chronic tendon and ligament diseases are commonly encountered in both athletic humans and animals, especially horses. Distal limb diseases, including suspensory ligament (SL) pathology due to anatomical, histological, and biomechanical properties, can be considered a model for tendon and ligament pathologies in humans. The appropriate selection of therapy is often crucial in optimising the healing process. One decisive factor influencing the possibility of returning to pre-disease training levels appears to be the utilisation of physical activity, including controlled movement, during the rehabilitation process. In the pathogenesis of musculoskeletal diseases and rehabilitation, adipocytokines play diverse roles. However, it is unclear what significance they hold in horses and in specific disease entities as well as the consequences of their mutual interactions. Recent studies indicate that in the pathogenesis of diseases with varied aetiologies in humans, their value varies at different stages, resulting in a diverse response to treatment. The results of this study demonstrate lower resistin concentrations in the venous blood plasma of horses with proximal suspensory desmopathy (PSD), while higher levels were observed in regularly trained and paddocked animals. The horses investigated in this study showed higher concentrations of resistin and IL-8, particularly in paddocked horses as well as in the working group of horses. The results suggest that these concentrations, including resistin in blood plasma, may be clinically significant. This attempt to explore the aetiopathogenesis of the processes occurring in the area of the proximal attachment of the suspensory ligament may optimise the procedures for the treatment and rehabilitation of horses.

## 1. Introduction

**Figure 1:**
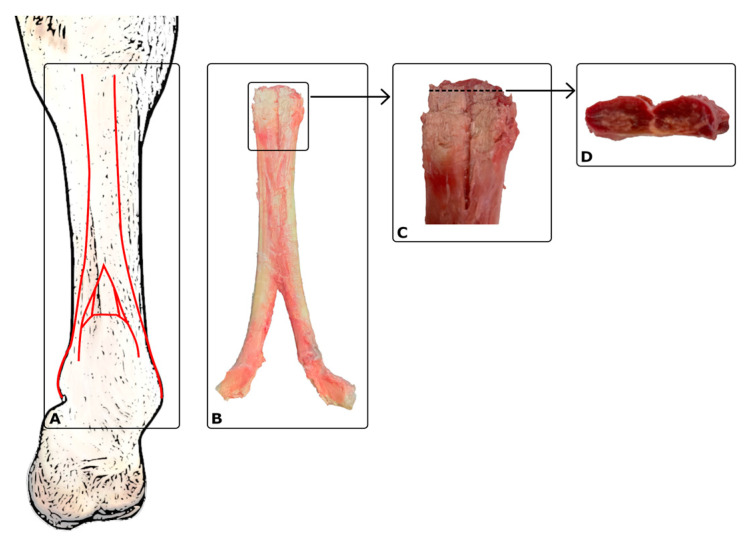
(**A**) Diagram of the suspensory ligament in a front limb—red lines; (**B**) suspensory ligament, dorsal view (dissected postmortem); (**C**) proximal suspensory ligament, dorsal view; (**D**) proximal suspensory ligament, cross-section (left side—medial lobe; right side—lateral lobe).

Diseases of the suspensory ligament, e.g., proximal suspensory desmopathy (PSD), are the most common causes of lameness in working horses. The suspensory ligament takes the form of a strong tendon band with elements of muscle and fat tissue as a result of the conversion of the limbs to the loads occurring during movement. The biomechanical relationships determine its susceptibility to overload and, as a result, damage [1]. In the vicinity of the proximal suspensory ligament (PSL), the area occupied by adipose tissue and connective tissue constitutes approximately 60–70% of each of the lobes, with the muscle cell compartment ranging from 20 to 40% [2]. The area and shape are individual features of each animal and can be compared to human fingerprints (Figure 1). Studies have demonstrated the presence of a rich intramedullary microvasculature in all areas of the suspensory ligament [3].

**Figure 2:**
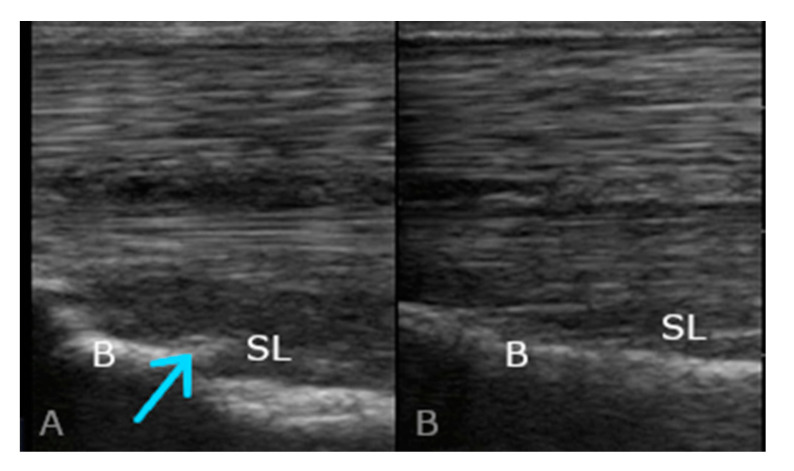
Longitudinal ultrasonographic image (proximal is to the left); (**A**) example of changes characteristic of SL desmopathy in the area of the proximal insertion of the interosseous muscle (PSL); (**B**) ultrasonographic image without PSD changes. B, bone surface; SL, proximal suspensory ligament, arrowhead, region of PSD changes in the PSL area.

Proximal suspensory desmopathy in horses affects both thoracic and pelvic limbs and can occur unilaterally or bilaterally. It may take an acute or chronic form. The most common clinical signs of suspensory ligament diseases, including PSD, are lameness, poor performance, and behavioural problems [1,4]. The majority of horses with chronic forms of PSD, both in the thoracic and pelvic limbs, exhibit subtle clinical symptoms, while chronic inflammatory conditions or repetitive injuries can lead to changes in the tissue structure, including the bones. Sudden trauma and the associated acute inflammatory state often take on a chronic form, becoming a potential source of pain. Identifying it can be challenging due to the frequent adaptations and compensations within the musculoskeletal system. Lameness, characterised as an alteration in the animal’s gait, is a clinical symptom resulting from pain perception. The people who look after horses often inadequately recognise lameness and pain, leading to delayed treatment initiation, thereby reducing the chances of full recovery and a return to the pre-disease level of training [4]. PSD often has no expressed local clinical symptoms, and the diagnosis depends on the response to local analgesia as well as ultrasound and X-ray changes [5,6]. The ultrasound image assesses the shape and size of SL, structure patterns of the fibres, echogenicity, and the bone surface in the PSL area. Often, patients with chronic lesions of the fatty lobes of the proximal suspensory ligament (medial and lateral) are characterised by a changed image of the structures and echogenicity in ultrasound images [7] (Figure 2). MRI studies have shown a correlation of the images for adipose tissue and muscle tissue with disease progression at the proximal attachment of the SL. The presence of adhesions has also been demonstrated as well as changes in the vascularisation and innervation of the area. Loss of muscle fibres and adipose tissue has also been reported in association with extensive histological abnormalities of the collagenous tissue, including areas of hemosiderin deposition, necrosis, collagen hyalinisation, cartilage and cartilage metaplasia, neovascularisation, and fibrosis [2,8].

In field conditions, there are clinician limitations in radiological imaging (e.g., ultrasound and X-rays). Complications can arise from the varying levels of veterinary expertise, subjective assessments during the clinical examinations, and the relatively short time frame within which a definitive diagnosis is expected [5].

There are anatomical and biomechanical similarities between the Achilles tendon in humans and the suspensory ligament in horses. The tendon in Achilles tendinopathy is described as macroscopically thickened, with an uneven brown coloration. Imaging shows a histologically increased tenocyte number and basal substance glycosaminoglycan concentration, collagen disorganisation and fragmentation, and neovascularisation without the presence of macrophages, neutrophils, or other inflammatory cells [9]. Healthy tendons or ligaments are relatively avascular. Neovascularisation is as one of the features of tendinopathy [10]. It is known that the peroneal adipose tissue and Kager’s adipose body within the Achilles tendon and, similarly, Hoffe’s subacromial adipose body share a common vascularisation with the surrounding tissue structures [11]. The associated flow of substances secreted by the adipose tissue may be related to the delivery of local and systemic adipocytokines and over time may influence local degenerative processes [12].

It seems interesting to determine whether the changes identified in the ultrasound, occurring locally within the PSL in horses in the case of desmopathy, may affect the concentration of selected adipokines in the peripheral blood plasma.

### Adipocytokines in Musculoskeletal Diseases

The adipokines (resistine, visfatine, and leptine) and IL-8 are produced exclusively by the adipose tissue and many other tissues [13]. The expression of adipokines has been reported in the circulated blood, synovial fluid, joint synovial membrane, fat body of the Achilles tendon, and Hoffa of the knee joint in humans as well as degeneratively altered articular cartilage [11,14]. The causal relationship between the development and progression of osteoarthritis of the knee and the adipose tissue was considered for the knee region through extensive interactions with the synovial membrane, articular cartilage, and subchondral bone [15].

The mechanisms of resistin’s biological effects are only partially understood. The action of the adipokine appears to be reversible and dependent on its concentration and conformation. They influence the interaction in terms of the physiological and pathological processes within individual cells and tissues [16]. By antagonising the insulin action, resistin reduces the glucose uptake in adipocytes, muscle cells, and other tissues, potentially acting as a small auxiliary chaperone [17]. A correlation has been observed between diverse training and its intensity and the concentration of resistin in the blood of humans and animals including horses [18,19].

Visfatin is known as nicotinamide phosphoribosyltransferase (NAMPT). The homology of the highly conserved protein sequence in multiple species (94% in mammals), such as humans, mice, and dogs, suggests an important role for this adipokine [20]. It functions as an immunomodulatory and pro-inflammatory cytokine, stimulating monocytes to produce pro-inflammatory cytokines including IL-8 and TNF-α [21]. The activity of visfatin is associated with the generation of NAD+, a fundamental energy and signalling molecule in most eukaryotic and prokaryotic organisms [22]. Visfatin participates in angiogenesis and may play a reparative role for endothelial cells [23]. The role of visfatin in musculoskeletal diseases in humans has been described [24]. In horses, its correlation with training methods has also been established [25].

Leptin is a multifunctional protein hormone, and its receptors have been identified in degenerated regions of both rat and human nucleus pulposus [26]. The bidirectional relationship of this adipokine within bone tissue is known as well as its connection to the regulation of bone mass and its involvement in the metabolic substrate reactions of the adipose tissue in the bone marrow [27] and myocardium [28]. Leptin induces the expression of matrix-degrading peptides and activates nitric oxide synthesis. It facilitates the activation of macrophages, neutrophils, dendritic cells, and NK cells, contributing to the development of an inflammatory environment in osteoarthritis joints [29]. The influence of leptin on nitric oxide (NO) production in relation to degeneration and the pathophysiology of lumbar spine pain in humans is also well known [30]. The increase in leptin concentration in the plasma of horses after training positively correlates with cortisol concentration in the plasma and occurs only during prolonged, moderately intense physical exertion [31].

IL-8 is a chemokine with an affinity for specific cellular receptors. Receptors in the endothelial and vascular cells have been observed to, when combined with IL-8, induce the secretion of vascular endothelial growth factor (VEGF), which stimulates angiogenesis [32]. In addition to adipose tissue, skeletal muscles have been recognised as an endocrine organ, where their cells produce cytokines with potential hormonal effects. After exercise, the increased expression of IL-8 in the muscle tissue is similar among different species, but its function has not been clarified well [33]. During physical training, increases in plasma cytokines (myokines) including IL-6 and IL-8 have been observed in humans in response to concentric exercise [34]. Exercise-induced inflammatory responses are thought to be required for the regenerative and adaptive processes of the musculoskeletal system [35].

The relationship between the involvement of individual proximal suspensory ligament adipose, muscle, and collagen tissues and the concentration of selected adipocytokines in venous blood appears insufficiently clear.

Based on reports in the literature, a potential relationship between local adipose tissue, vascularisation, and the development of inflammation, degeneration, and pain sensation in diseases of proximal origin concerning suspensory ligament attachment has not been described. As chronic SL conditions including PSD lead to a change in the proportion of adipose tissue in favour of connective tissue, such an effect may influence the levels of secreted adipocytokines locally and systemically, including those analysed in the presentation of this study.

## 2. Results

### 2.1. Parameters of Clinical Characteristics

In the group of horses diagnosed with desmopathy of proximal suspensory desmopathy, there were twice as many geldings as mares, with a similarly higher proportion of males in the control group. The Definitions of terms used to characterise the group list in Table 1. A comparison of the age of the horses between the group of animals with PSD and the control group showed a statistically significant difference (U = 56, *p* = 0.03) (Table 2). In horses diagnosed with PSD, lameness of the thoracic limbs was reported in 85% of cases (of which 44% PHL, 41% HPL) (Table 3).

### 2.2. Ultrasound Examination

The ultrasound image of PSL damage in the acute phase, as a result of inflammation, swelling, and disruption of collagen fibres, is hypoechoic. The next stage—the proliferation phase—includes angiogenesis and fibroblast infiltration, which results in increased echogenicity. Ultrasonographically, the size of a naturally occurring acute lesion typically increases during the first 2 weeks after injury and then gradually decreases during healing. The study took into account changes over 3 weeks from the discovery of lameness as a clinical symptom recorded and reported by the animal’s owner.

**Figure 3:**
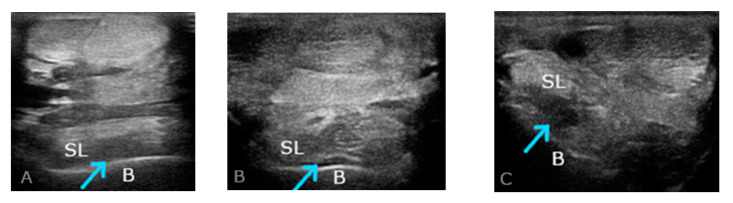
Transverse plane ultrasonographic images of proximal suspensory desmopathy (PSD); (**A**–**C**) the images show heterogeneous changes, mainly hypoechoic of SL (suspensory ligament) in chronic phase (>3 weeks). (**A**) The weightbearing limb examined; (**B**) the examined limb unloaded; (**C**) medial view: the examined limb unloaded, showing enlargement of medial lobe of SL. Only plantar (**B**) and medial (**C**) views allow more precise imaging of the lobes of SL. A normal ultrasound image of SL shows a well-defined structure with a homogeneous, mainly isoechoic fibre pattern. SL, suspensory ligament; B, third metacarpal bone; arrowhead, region of PSD changes in the PSL area.

**Figure 4:**
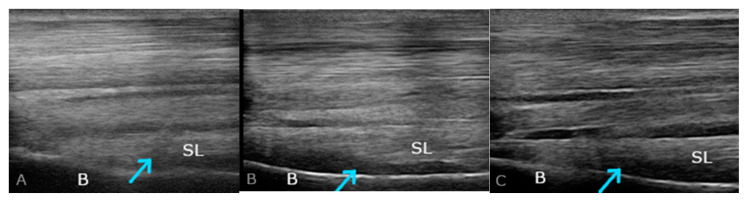
Longitudinal ultrasonographic images of changes indicative of proximal suspensory desmopathy (PSD); the weightbearing limbs were examined; (**A**–**C**) the images show heterogeneous changes, mainly hypoechoic of SL (suspensory ligament) in chronic phase (>3 weeks). A normal ultrasound image of SL shows a well-defined structure with a homogeneous, mainly isoechoic fibre pattern. SL, suspensory ligament; B, third metacarpal bone; arrowhead, region of PSD changes in the PSL area.

Ultrasonographic evaluation of the proximal suspensory ligament (PSL) attachment was performed in the transverse and longitudinal planes. Using the assessment of PSL, the elevated limb (Figure 3B,C) and with the limb loaded (Figure 4) were considered. Consistent imaging allowed for the assessment of size, shape, margins, echogenicity, and changes in the bone surface. Conducting a detailed interview with the owner regarding the animal’s use and history as well as an analysis of clinical symptoms and radiological imaging allowed the diagnosis of proximal suspensory desmopathy (PSD) to be made.

### 2.3. Relationship between the Concentration Levels of the Studied Cytokines and Clinical Characteristic Parameters—Laboratory Studies

The correlation between the level of resistin concentration and clinical characteristic parameters was investigated in the whole group of horses as well as in the subdivided subgroups, taking into account the diagnosis of the proximal suspensory ligament desmopathy.

The analysis revealed a significant and strong correlation between resistin concentration and the parameters of the leukocyte system (WBC, neutrophils, lymphocytes, NLR, and MLR) in the group of horses diagnosed with PSD. A positive correlation was also observed between resistin concentration and WBC as well as neutrophil values for all animals (Table 4). No relationship was found between measured calcium, sodium, and potassium ions in any of the groups or for the studied adipocytokines.

### 2.4. Impact of Desmopathy of the Proximal Suspensory Ligament on Resistin, Wisfatin, Leptin, and IL-8 Concentration in the Blood Plasma

The Comparison of Concentrations of the Studied Adipokines and IL-8 in the Study Group with Confirmed PSD Diagnosis and the Control Group without Musculoskeletal Disorders (C).

**Figure 5:**
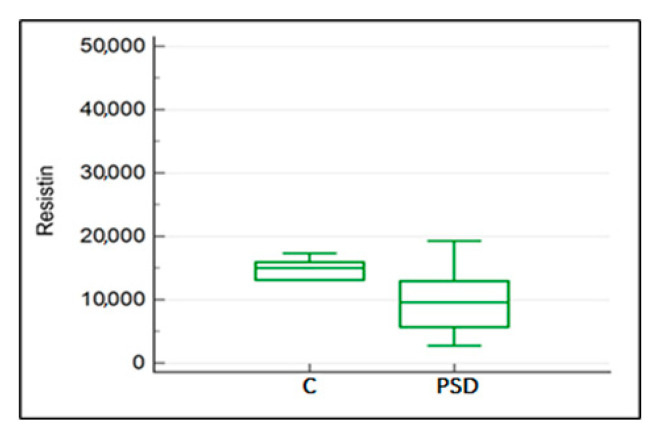
The comparison of resistin concentrations between the study group with confirmed PSD diagnosis and the control group without musculoskeletal disorders (C).

Statistical analysis revealed a significant difference in resistin levels between the groups with PSD diagnosis and the control groups (C) (U = 56, *p* = 0.027). The median for horses in the control group was 14,981.71 ng/mL, while the median for horses diagnosed with PSD was 9565.04 ng/mL (Figure 5).

**Figure 6:**
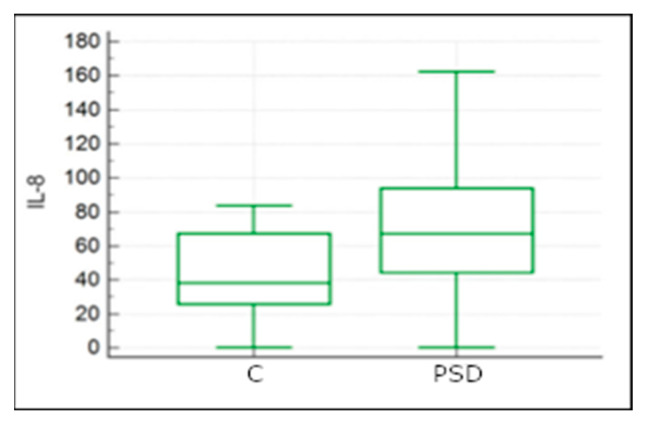
The comparison of IL-8 concentrations between the study group with confirmed PSD diagnosis and the control group without musculoskeletal disorders (C).

In the statistical analysis, a significant difference in IL-8 concentration levels between the PSD and C groups was observed (U = 57, *p* = 0.04). The median in the healthy horse group was 38.27 ng/mL, while in the group with diagnosed proximal suspensory desmopathy, it was 67.14 ng/mL (Figure 6).

### 2.5. Impact of the Activity Musculoskeletal System on Resistin, Wisfatin, Leptin, and IL-8 Concentration in the Blood Plasma

**Figure 7:**
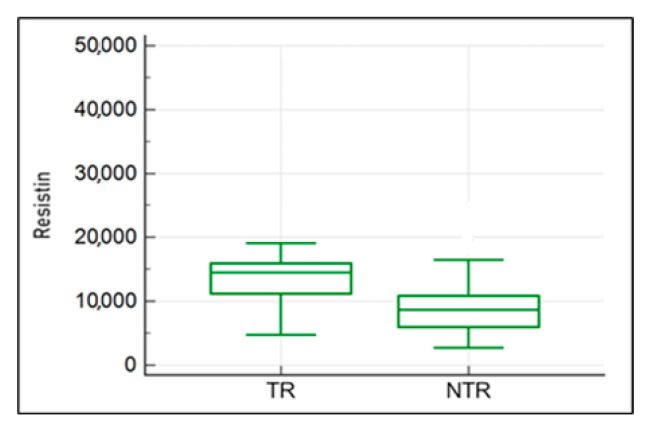
The comparison of resistin concentrations in the blood plasma of horses in the TR and NTR groups. TR, regularly working or training horses; NTR, horses excluded from work.

The comparison of concentrations of the studied adipocytokines in all horses included in the study (without division into healthy and diseased) revealed higher resistin levels in the plasma of regularly working and training horses (U = 73, *p* = 0.016) compared to horses excluded from work (Figure 7). Similarly, in the group of horses with PSD, the analysis showed a similar trend, but the difference was not statistically significant (TR—14,506.8 ng/mL; NRT—8642.21 ng/mL).

**Figure 8:**
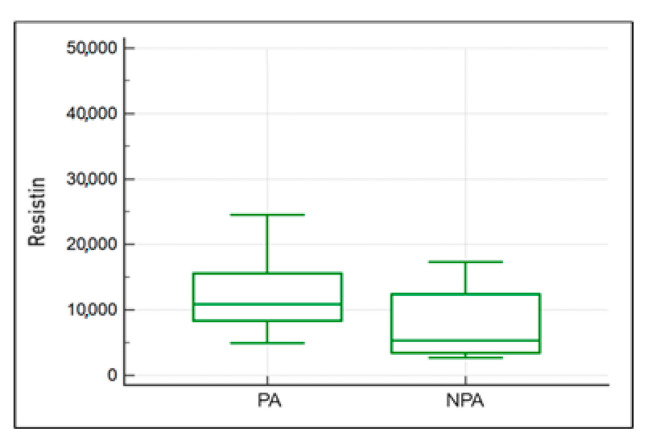
The comparison of resistin concentrations in the blood of horses in the PA and NPA groups. The comparison of concentrations of studied adipokines and IL-8 in the paddocked (PA) and non-paddocked (NPA) horse groups.

The comparison of concentrations of studied adipokines and IL-8 in the paddocked (PA) and non-paddocked (NPA) horse groups revealed the medians of PA (10,914.8 ng/mL) and NPA (5299.3 ng/mL), respectively (U = 50, *p* = 1.89) (Figure 8). A similar comparison was conducted in the group of horses with diagnosed PSL desmopathy, showing a similar trend, i.e., higher resistin levels in paddocked animals: for PA 10,543.56 ng/mL and for NPA 4695.69 ng/mL. The trend was not statistically significant.

### 2.6. Correlations between Leptin Concentration and Resistin, Visfatin, and IL-8 Concentrations in the Blood Plasma among the Isolated Groups: Study Group (PSD) and Control Group (C)

A strong positive correlation was obtained between leptin concentration and visfatin concentration in the group of all examined horses. In the group of horses diagnosed with proximal suspensory desmopathy, a strong positive correlation was found between leptin and visfatin levels. Additionally, a strong positive correlation was observed between leptin concentration and IL-8 level in the group of healthy horses (Table 5).

No significant correlations were found with the other adipokines.

## 3. Discussion

Early diagnosis of diseases of tendons or ligaments is essential for effective treatment results in humans and animals. In the available literature, both tendinopathy and desmopathy in horses are well described as common injuries that can lead to the end of an athletic career [36].

There are no reports in the available literature on the association between musculoskeletal diseases including that of the suspensory ligament and venous plasma adipokine concentrations in horses. Therefore, the following discussion refers to the literature on the changes in blood concentrations of selected adipokines and IL-8 in humans and various animal species as well as to thematically related disease entities.

The studies conducted on humans and animals, including dogs, in diseases of the musculoskeletal system take into account the possible impact of locally occurring disease entities on the concentration of adipokines in the peripheral blood plasma [27,29,30].

In both cases, the interactions of external and internal factors and the biomechanical and histological characteristics of the structures and their surrounding areas are taken into account in the disease processes [37,38].

Dyson et al. stated that in cases of PSD, the pain and resulting lameness can have a variety of causes [38]. In their work, Docheva et al. confirmed that the sources of pain in Achilles tendinopathy in humans are also complex. As examples, they mentioned increased prostaglandin production in the matrix, neovascularisation, changes in tenocyte structure and function, and changes in metabolite concentrations [39].

At the cellular level, Durgam et al. identified the non-neuronal cholinergic system as a pain factor. Chemical irritants were also proposed, such as cytokines, tumour necrosis factor alpha, interleukins, signalling molecules (Ca^2+^ and adenosine triphosphate), neuropeptides (substance P and neuropeptide Y), and neurotransmitters (glutamate) [40]. In addition, Scheller et al. considered mechanoreceptors and nerve-related components such as N-methyl-D-aspartate glutamate receptors (NMDA), which are involved in tendinopathy via the blood vessels present [41]. Benjamin et al. identified a potential source of pain in the repetitive stress-inducing effect on the fat body located near the tendon structures in the Achilles attachment area. Presumably, the resulting chronic low-grade inflammation negatively affecting the nerve endings located in the adipose tissue leads to pain [15]. Potentially, the adipose tissue present in the suspensory ligament in horses can be viewed in a similar way, both innervated and vascularised, perhaps as a source of adipocytokines and pain. Future research in this area may confirm a similar aetiology of pain-related processes often clinically expressed as lameness in the animal [42].

The peroneal adipose tissue and Kager’s adipose body within the Achilles tendon and, similarly, Hoffe’s subacromial adipose body share a common vascularisation with surrounding tissue structures [11]. The associated flow of substances secreted by adipose tissue may be related to the delivery of local and systemic adipocytokines and over time may influence local degenerative processes [12]. As described by de Casto Pochini, the innervation of the Achilles tendon attachment area located in the adipose tissue suggests their mechanosensory role. Disease processes in this area can lead to the proliferation of blood vessels and nerves, causing pain within the lesions [43]. Similar anatomical–functional relationships have been observed for the suspensory ligament in horses, which was compared in publications by Crass et al. and Gilles et al. to the Achilles tendon [37,44]. Pochini et al. found that the Achilles tendon and adjacent Kager’s adipose body and Hoffe’s hypopharyngeal adipose body are sites of adipocytokine secretion [43,45,46]. 

Recent studies have described the role of resistin in humans in stress biology, including as a diagnostic biomarker to assess disease and treatment outcomes. It has been confirmed that resistin is involved in the secretion of immune effectors and drives diverse physiological functions through interleukin IL-8 and IL-6, among others, which stimulate a proinflammatory response [47]. In the group of diseased horses presented in the results, this study showed a correlation between reduced resistin levels and elevated IL-8 and white blood cell system values. In their study, Calabro et al. found that in humans and animals, resistin triggers smooth muscle proliferation in the blood vessels [48]. Verema et al. and Kougias et al. described its effect on vascular endothelial dysfunction [49,50]. A complementary reference is the work of Cho et al., where they indicated the promotion of endothelial infiltration and adhesion by monocytes [51]. In our study, routine morphology and biochemistry examinations were performed. In the group of horses diagnosed with PSD, the analysis revealed a significant, strong correlation between resistin concentration and the parameters of the leukocyte system (WBC, neutrophils, lymphocytes, NLR, and MLR). For all groups of animals, it has been observed that there is a positive correlation between resistin concentration and WBC as well as neutrophil values. In the case of measured calcium, sodium, and potassium ions, no relationship was found in any of the groups or for the studied adipocytokines. 

The relationship between the concentration of white blood cells and the tested adipokines in horses is currently unknown.

It is possible that a similar relationship exists for changes in the endothelial vascularisation of the proximal attachment of the suspensory ligament in horses, including its fat pads and other tissues of the PSL.

This would need to be verified in further research conducted in the local SL environment.

Steppan et al. showed that the resistin gene (Retn) is mainly expressed in the adipocytes of the adipose tissue and white blood cells [52].

Scientific observations have shown a correlation between resistin and exercise. Fatouros et al. highlighted the relationship between training intensity and frequency in humans and markers of inflammation [53]. In contrast, experiments by Lavie et al. and Van Pelt et al. showed in humans an inverse relationship between physical activity and low-grade inflammation [54,55]. Ihalainen et al. described the effect of high-intensity training, which induces an increase in selected inflammatory biomarkers and a reduction in certain inflammatory cytokines as well as leptin and resistin [56].

In the results presented in this paper, higher concentrations of resistin were obtained in the venous blood plasma of horses that worked regularly compared to horses that did not, taking all horses included in the study into account.

Nikiforov et al. described the enzymatic activity of visfatin as nicotinamide phosphoribosyltransferase (NAMPT) [57]. In our own studies on horses, we did not observe significant differences in the concentration of visfatin in venous blood plasma between the group of horses with PSD and the control group.

Kędzierski et al. examined the concentration of visfatin in the peripheral venous blood of racehorses undergoing training. It was demonstrated that short-term, intense exercises performed during race training had no impact on the levels of the investigated adipokine [25]. In our own studies, we also did not observe differences in the concentration of visfatin in the peripheral venous blood, considering horses in training cycles (working) and non-working ones as well as those maintained in a paddock system.

The expression of IL-8 protein in the skeletal muscle fibres during post-exertional regeneration strongly suggests that exercise can stimulate the muscle cells to produce IL-8. It is speculated that inflammatory reactions induced by physical exertion are required for the regenerative and adaptive processes of the musculoskeletal system. It is also known that the induction of cytokine expression may play an important role in regenerating and counteracting inflammation in the skeletal muscle tissues in horses after exercise. The induction of cytokine expression may play a significant role in regenerating and counteracting inflammation in the skeletal muscle tissues in horses after exercise. During physical exercise in humans, an increase in cytokine values, including IL-6 and IL-8 in the plasma, has been described in response to training [58]. In a study of thoroughbred horses, the level of myokines, including IL-8, increased after training. Higher levels of IL-8 were observed in our study in regularly trained animals. A similar trend was visible in paddocked horses. A higher cytokine value was observed in these groups, both in the subgroup of healthy horses and those diagnosed with PSD. The analysis of the results of this study revealed higher IL-8 levels in the plasma of horses with PSD. However, no differences were observed regarding the way horses were kept in both the subgroups of healthy and diseased horses.

The individual impact of leptin in combination with inflammatory mediators significantly accelerates the production of nitric oxide (NO) [59]. This likely relates to degenerative conditions in humans, particularly in lumbar spine pain [60]. The literature provides ambiguous research results; some research suggests the proinflammatory implications of leptin in the pathogenesis of Achilles tendon tendinopathy [61,62]. Rechardt et al. suggested a reduced chance of recovery for patients with upper-limb soft tissue diseases who have a higher level of leptin in the plasma. The reasons are attributed to the antagonistic actions of TGF-b against resistin and visfatin [63]. Patients with degenerative joint disease, when exposed to leptin, respond by producing proinflammatory cytokines such as IL-8, IL-6, and TNF-alpha, which may intensify the catabolism of proteoglycans, increase the expression of metalloproteinases (MMP), and thereby stimulate cartilage degradation. Sui et al. did not observe similar processes in healthy individuals [62]. In studies on patients with rheumatoid arthritis (RA), a hypothesis was proposed regarding a possible increase in the production of IL-8 and IL-6 induced by leptin [64].

Observations made in our study did not confirm a relationship between the levels of visfatin, resistin, and IL-8 in all groups of horses. However, a strong correlation between visfatin and leptin was demonstrated in the group of horses with PSD. In the analysed group of healthy horses, a strong positive correlation occurred between the leptin concentration and the interleukin-8 value.

Kędzierski, in his study, examined the impact of training on the concentration of leptin in the plasma of sport horses. It was observed that in horses starting their racing career, training led to a decrease in leptin levels. In contrast, in horses trained for several seasons, the leptin levels in the plasma remained unchanged. A significantly interesting observation was the relationship between behavioural problems and low leptin levels in young horses participating in intensive training [65]. In the present study, no correlation was found between the concentration of leptin and the presence of training or paddock maintenance in either healthy (C) or diseased (PSD) horses.

The available literature shows that resistins as well as other adipocytokines cannot be seen as involved in single disease processes but as molecules with diversified physiological functions [16,66].

## 4. Materials and Methods

Twenty-seven horses were diagnosed with proximal suspensory desmopathy and the resulting lameness (musculoskeletal movement disorder), while the control group (C) consisted of nine horses without lameness and with no pathological movement disorders with regard to the musculoskeletal system. Both groups of horses were patients of the Department of Animal Surgery at the University of Life Sciences in Lublin. The study group (PSD) comprised 27 animals. The animals for the test group were qualified based on the following criteria: lameness lasting no shorter than 21 days, PSD diagnosis, age of the horse between 4 and 20 years, a BCS range of 2–3 (Body Condition Scoring of Horses), no symptoms of comorbidities, and no treatment in the 3 months preceding the study. Animals for the control group were qualified based on the following criteria: absence of a diagnosis of PSD and lameness after an examination performed for routine control, prophylaxis, or clinical check-up with the owner’s willingness and knowledge as well as age of the horse between 4 and 20 years, a BCS range of 2–3 (Body Condition Scoring of Horses), no symptoms of comorbidities, no treatment in the 3 months preceding the study, and informed consent of the owner for participation in the study. To minimise the influence of factors that could potentially affect the levels of the adipokines studied, the following exclusion criteria were applied: grades 0–1 and 4–5 BCS, presence of other diagnosed musculoskeletal injuries, respiratory diseases, rutting season, and sex of the horse—stallion.

### 4.1. Scheme of the Study Group Eligibility Test

The eligibility study for the study group included the following: a detailed medical and veterinary history of the symptoms, maintenance methods, feeding, use and medical history of the animal, and clinical examination including BCS assessment, imaging testing, and laboratory tests.

### 4.2. Veterinary Medical History, Clinical and Imaging Studies

In order to reduce the number of variables that could affect the outcome of the clinical examination and to maintain similar examination conditions, all stages of the examination of every horse were performed by a single person, the author of this paper, with a level of training and experience appropriate for making a correct diagnosis (DVM, Ph.D.). Orthopaedic examination of the animal “standing up” and evaluation of the horse’s condition (muscling and fatness) were performed based on the Body Condition Score (BCS). The BCS was used to determine the body fat content of the study horses. The results were averaged to obtain an overall measurement [67,68]. The data for horses with excessive body fat were not qualified for further analysis.

A study of the animal in motion was performed as follows: straight tarsus test on firm ground, straight trot test on firm ground, a tarsus test on wheels on soft ground, trot test on wheels on soft ground, and examination under saddle if further diagnosis was required. 

The assessment of lameness in each horse was carried out on a six-point scale, which was based on observations obtained during the clinical examination of the horse in motion according to the protocol outlined above (Lameness Grading System: American Association Equine Practitioners). Functional flexion tests of the thoracic and pelvic limbs were performed on each test animal. The degree of lameness during flexion tests was assessed. Diagnostic anaesthesia was performed if the doctor considered it necessary in order to rule out or confirm the pain in the area. Identification of SL lesions was performed using ultrasonography, with determination of the location in transverse and longitudinal projections on the weight-bearing limb. Structures were also imaged on the limb raised and flexed dorsally at 30 degrees [69]. 

### 4.3. Laboratory Analysis

The material for the study consisted of 10 mL of peripheral blood collected from the external jugular vein into tubes containing EDTA during routine examinations. The blood samples were then subjected to laboratory processing: cooling to 4 °C and centrifugation for 10 min at 1500 rpm. The resulting plasma was collected, portioned, and stored at −80 °C until further indication.

### 4.4. Method Used to Assess Adipokine and Interleukin 8 Levels

Levels of leptin, resistin, visfatin, and interleukin 8 were assessed using a magnetic bead-based method with commercially available Multiplex kits and the MagPiX automatic analyser from Luminex^®^. A commercially available Multiplex kit for determining the test substances was used, and after consultation with the kit manufacturer, a comparison of the sequences of the tested cytokines in horses and humans was made, and a satisfactory level of homology was found. After analysing the results obtained for selected substances in the blood of horses, it was decided to use the Multiplex kit to determine the levels of the cytokines studied and IL-8 in the presented study.

### 4.5. Experiment Process

Plasma samples were diluted as appropriate and added to microspheres coated with capture antibodies that sequentially bound to the corresponding substances, allowing them to be marked. In the next step, the mixture was supplemented with biotinylated detection antibodies binding to the specific markers to be determined. Concentrations of selected adipokines and interleukin 8 were assessed according to the manufacturer’s protocol. Concentration calculations were performed according to the five-parameter standard logarithmic curve using the xPonent 4.2 software.

### 4.6. Statistical Analysis

The data obtained from the study were subjected to descriptive and comparative statistical analysis. The Shapiro–Wilk test was used to analyse the normal distribution of the study variables. The assessment of the significance of differences between samples depending on the distribution of the variables studied and the number of groups compared was based on parametric and non-parametric statistical tests. A Student’s *t*-test was used to compare variables with a normal distribution. The Mann–Whitney test was used to compare variables noncompliant with the normal distribution. In all correlation analyses, it was necessary to use Spearman’s non-parametric rank test due to the lack of normal distribution of one or both correlated variables. The statistical analysis was performed using the MedCalc v.22.016 software. The analysis assumed a 5% risk of inference error, and results with a *p*-value less than or equal to 0.05 were considered statistically significant. The results are presented in the form of tables (detailing statistically significant relationships between study groups) and figures (for significant results).

## 5. Conclusions

The exact significance of adipokines in specific disease entities and the consequences of their mutual interactions remain unclear [70].

The available literature shows that adipocytokines cannot be seen as involved in single disease processes but as molecules with diversified physiological functions [16,66].

Based on our own research and literature data, the full role of adipokines in the pathogenesis of PSD in horses and the consequences of their mutual interactions have not been fully explained. PSD is a disorder with a multifactorial aetiology; therefore, the search for and attempts to identify new markers involved in the pathogenesis of the disease may contribute to the introduction of preventive measures and the modification of treatment and rehabilitation methods, taking into account the physical activity. The studies were preliminary, so it is necessary to expand the research profile and longitudinally observe the action of adipocytokines, especially in the local environment of the proximal suspensory ligament. When assessing the concentration of selected adipokines, it seems appropriate to take into account the intensity of physical activity of the animals in order to qualify them for the analysed research groups.

An interesting and planned direction for future research is the measurement of local adipokine concentrations in the area of the proximal attachment of the suspensory ligament and histology of altered tissues in relation to the imaging studies.

### Limitations of the Study

Logistic and financial constraints limited the following:

1. Extended radiological diagnostics using magnetic resonance imaging;
2. Supplementing with additional markers including tumour necrosis factor, interleukin-6, and vascular endothelial growth factor.

## Figures and Tables

**Table 1:** Definitions of terms used to characterise the group.

Term	Definitions of the Characteristic Terms Used
Lameness	A change in the horse’s gait, usually caused by pain or mechanical restriction.
Chronic condition	Disease symptoms that may occur with PSD, including but not limited to lameness, lasting no less than 21 days.
Maintenance method	Determines the conditions of the horses’ daily living, i.e., whether they had access to a paddock and the time they were out of the stable.
Paddocking	This means that the horse has been in the paddock, regardless of its surface area, at least once a day for a minimum of 2 h at least 4 days a week.
No paddocking	This means that the conditions for keeping the horse did not meet the criteria given for the term “paddocking”; leaving the stable only for training, walking in hand, and/or using the carousel qualified as “no paddocking”.
Method of use	Defines the conditions of use of the horse by the rider, i.e., regular work under the saddle; used for recreational riding or sporting recreation.
Training	Daily work under the saddle, lasting a minimum of 40 to 60 min a day, at least 4 times a week, regardless of the riding discipline and the level of training of the horse and rider.
No training	This means that the conditions of use of the horse by the rider did not meet the criteria given for the term “training”.

**Table 2:** Characterisation of the animals under study.

Feature	PSD Group		Control Group	
	n	(%)	n	(%)
Gender mare	7	25	2	22
Gelding	14	53	7	78
Stallion	6	22	-	-
Age (years)	13		7	
median	(9–20)		(4–18)	
Breed—half-bred horse	25	93	7	78
Wielkopolska	2	7	-	-
Other	-	-	2	22
BCS 2	22	81	5	55
BCS 3	7	19	4	45

BCS, body condition scoring—an objective system for assessing the horse’s condition level (amount of stored fat and muscle development) by numerical assessment on a scale from 1 to 5.

**Table 3:** Characterisation of horses in the study group (PSD) and the control group (C) considering inclusion features.

History	PSD Group n %		Control Group n %	
Lameness	27 (100%)		0	
Right front limb	12	44	-	-
Left front limb	11	11	-	-
Right hind limb	1	4	-	-
Left hind limb	3	11	-	-
Housing method (all horses)	36 (100%)			
Paddock	22	61	6	26
No paddock	5	14	3	8
Usage method (all horses)	36 (100%)			
Training (working)	18	50	6	17
No training (non-working)	9	25	3	8

**Table 4:** Comparison of selected morphological parameters of blood in the groups.

Resistin	All Horses		PSD		C	
	r	*p*	r	*p*	r	*p*
Age	0.12	0.50	0.29	0.15	0.38	0.32
RBC	0.09	0.63	0.12	0.95	0.33	0.38
HGB	0.12	0.52	0.15	0.47	0.37	0.33
MCH	0.44	0.01	0.45	0.03	0.01	0.98
MCHC	0.43	0.01	0.30	0.14	0.14	0.73
MCV	0.28	0.12	0.39	0.05	0.08	0.85
WBC	0.36	0.03	0.49	0.01	0.37	0.33
NEU	0.40	0.02	0.59	0.002	0.30	0.44
LIMF	0.04	0.84	0.44	0.03	0.47	0.21
NLR	0.28	0.11	0.50	0.01	0.10	0.79
MLR	0.21	0.23	0.41	0.04	0.03	0.93
PLT	0.02	0.89	0.001	0.99	0.35	0.36

Selected clinical parameters: PSD, group with confirmed PSD diagnosis; C, control group; r, Spearman’s rank correlation coefficient; *p*, significance level < 0.05.

**Table 5:** Correlations between leptin concentration and resistin, visfatin, and IL-8 concentrations in the group of all examined horses and subgroups based on a positive diagnosis of PSD (PSD) and the control group (C).

Leptin	n	All Horses (ng/mL)		n	PSD Group (ng/mL)		n	C Group (ng/mL)	
Resistin	36	r	0.15	27	r	0.15	9	r	0.024
		*p*	0.412		*p*	0.412		*p*	0.96
Visfatin	36	r	0.63	27	r	0.63	9	r	0.47
		*p*	0.001		*p*	0.001		*p*	0.24
IL-8	36	r	0.12	27	r	0.12	9	r	0.89
		*p*	0.58		*p*	0.58		*p*	0.003

PSD, group with confirmed PSD diagnosis; C, control group; n, number of individuals in a given group; r, Spearman’s rank correlation coefficient, *p*, significance level.

## Data Availability

The data presented in this study are available on request from the corresponding author.

## References

[1] Dyson S. (1994). Proximal suspensory desmitis in the hindlimb: 42 cases. Br. Vet. J..

[2] Nagy A., Dyson S. (2009). Magnetic resonance anatomy of the proximal metacarpal region of the horse described from images acquired from low- and high-field magnets. Vet. Radiol. Ultrasound.

[3] Williams M.R., Arnoczky S.P., Pease A.P., Stick J.A. (2013). Microvasculature of the suspensory ligament of the forelimb of horses. Am. J. Vet. Res..

[4] Dyson S., Van Dijk J. (2020). Application of a ridden horse ethogram to video recordings of 21 horses before and after diagnostic analgesia: Reduction in behaviour scores. Equine Vet. Educ..

[5] Hardeman A., Egenvall A., Braganca F., Swagemakers J.H., Koene M., Roepstorff L., van Weeren P., Byström A. (2022). Visual lameness assessment in comparison to quantitative gait analysis data in horses. Equine Vet. J..

[6] Dyson S., Pinilla M.J., Bolas N., Murray R. (2018). Proximal suspensory desmopathy in hindlimbs: Magnetic resonance imaging, gross post-mortem and histological study. Equine Vet. J..

[7] Werpy N.M. Review of Non-Weight-Bearing Proximal Suspensory Ligament Ultrasound for Alterations in the Muscle/Fat Indicating Pathologic Change. How to Maximaze the Use of Ultrasound in the Field. AAEP Proceedings, Volume 67, pp. 49–57. https://aaep.org/sites/default/files/2022-05/Werpy,%20Natasha.pdf.

[8] Denoix J.-M., Perrot P., Bousseau B., Sciciuna C. (1991). Images echographiques des lésions du muscle interosseux III (ligament suspenseur du boulet). Prat. Vét. Equine.

[9] Li H.Y., Hua Y.H. (2016). Achilles Tendinopathy: Current Concepts about the Basic Science and Clinical Treatments. BioMed Res. Int..

[10] Shukunami C., Takimoto A., Oro M., Hiraki Y. (2006). Scleraxis positively regulates the expression of tenomodulin, a differentiation marker of tenocytes. Dev. Biol..

[11] Pingel J., Petersen M.C., Fredberg U., Kjær S.G., Quistorff B., Langberg H., Hansen J.B. (2015). Inflammatory and Metabolic Alterations of Kager’s Fat Pad in Chronic Achilles Tendinopathy. PLoS ONE.

[12] Falcão-Pires I., Castro-Chaves P., Miranda-Silva D., Lourenço A.P., Leite-Moreira A.F. (2012). Physiological, pathological and potential therapeutic roles of adipokines. Drug Discov. Today.

[13] Pedersen B.K., Febbraio M.A. (2012). Muscles, exercise and obesity: Skeletal muscle as a secretory organ. Nature reviews. Endocrinology.

[14] Bonakdari H., Tardif G., Abram F., Pelletier J.P., Martel-Pelletier J. (2020). Serum adipokines/related inflammatory factors and ratios as predictors of infrapatellar fat pad volume in osteoarthritis: Applying comprehensive machine learning approaches. Sci. Rep..

[15] Benjamin M., Redman S., Milz S., Büttner A., Amin A., Moriggl B., Brenner E., Emery P., McGonagle D., Bydder G. (2004). Adipose tissue at entheses: The rheumatological implications of its distribution. A potential site of pain and stress dissipation?. Ann. Rheum. Dis..

[16] Codoñer-Franch P., Alonso-Iglesias E. (2015). Resistin: Insulin resistance to malignancy. Clin. Chim. Acta Int. J. Clin. Chem..

[17] Tripathi D., Kant S., Pandey S., Ehtesham N.Z. (2020). Resistin in metabolism, inflammation, and disease. FEBS J..

[18] Kędzierski W.K. (2021). Resistin concentration in blood plasma of racing and rally horses. Proceedings of the “Omnia Autem Animalia Sunt”: XVI Kongres Polskiego Towarzystwa Nauk Weterynaryjnych.

[19] Fuentes-Romero B., Muñoz-Prieto A., Cerón J.J., Martín-Cuervo M., Iglesias-García M., Aguilera-Tejero E., Díez-Castro E. (2021). Measurement of Plasma Resistin Concentrations in Horses with Metabolic and Inflammatory Disorders. Animals.

[20] McGlothlin J.R., Gao L., Lavoie T., Simon B.A., Easley R.B., Ma S.F., Rumala B.B., Garcia J.G., Ye S.Q. (2005). Molecular cloning and characterization of canine pre-B-cell colony-enhancing factor. Biochem. Genet..

[21] Garten A., Schuster S., Penke M., Gorski T., de Giorgis T., Kiess W. (2015). Physiological and pathophysiological roles of NAMPT and NAD metabolism. Nature reviews. Endocrinology.

[22] Revollo J.R., Grimm A.A., Imai S. (2007). The regulation of nicotinamide adenine dinucleotide biosynthesis by Nampt/PBEF/visfatin in mammals. Curr. Opin. Gastroenterol..

[23] Chiu C.Z., Wang B.W., Yu Y.J., Shyu K.G. (2020). Hyperbaric oxygen activates visfatin expression and angiogenesis via angiotensin II and JNK pathway in hypoxic human coronary artery endothelial cells. J. Cell. Mol. Med..

[24] Gosset M., Berenbaum F., Salvat C., Sautet A., Pigenet A., Tahiri K., Jacques C. (2008). Crucial role of visfatin/pre-B cell colony-enhancing factor in matrix degradation and prostaglandin E2 synthesis in chondrocytes: Possible influence on osteoarthritis. Arthritis Rheum..

[25] Kędzierski W., Janczarek I., Wilk I., Staniszewska M., Kowalik S. (2018). Plasma visfatin response to the intensity of exercise and training in race-horses. Pferdeheilkunde Equine Med..

[26] Sweeney G., Keen J., Somwar R., Konrad D., Garg R., Klip A. (2001). High leptin levels acutely inhibit insulin-stimulated glucose uptake without affecting glucose transporter 4 translocation in l6 rat skeletal muscle cells. Endocrinology.

[27] Rademacher J., Tietz L.M., Le L., Hartl A., Hermann K.A., Sieper J., Mansmann U., Rudwaleit M., Poddubnyy D. (2019). Added value of biomarkers compared with clinical parameters for the prediction of radiographic spinal progression in axial spondyloarthritis. Rheumatology.

[28] Abel E.D., Litwin S.E., Sweeney G. (2008). Cardiac remodeling in obesity. Physiol. Rev..

[29] Kleine S.A., Sanderson S.L., George C., Roth I., Gogal R.M., Thaliath M.A., Budsberg S.C. (2019). Correlation of serum and synovial leptin concentrations with body condition scores in healthy and osteoarthritic dogs. Vet. Surg. VS.

[30] Bas S., Finckh A., Puskas G.J., Suva D., Hoffmeyer P., Gabay C., Lübbeke A. (2014). Adipokines correlate with pain in lower limb osteoarthritis: Different associations in hip and knee. Int. Orthop..

[31] Kędzierski W. (2014). Changes in plasma leptin concentration during different types of exercises performed by horses. Anim. Int. J. Anim. Biosci..

[32] Heidemann J., Ogawa H., Dwinell M.B., Rafiee P., Maaser C., Gockel H.R., Otterson M.F., Ota D.M., Lugering N., Domschke W. (2003). Angiogenic effects of interleukin 8 (CXCL8) in human intestinal microvascular endothelial cells are mediated by CXCR2. J. Biol. Chem..

[33] Lee H.G., Choi J.Y., Park J.W., Park T.S., Song K.D., Shin D., Cho B.W. (2019). Effects of exercise on myokine gene expression in horse skeletal muscles. Asian-Australas. J. Anim. Sci..

[34] Chan M.H., Carey A.L., Watt M.J., Febbraio M.A. (2004). Cytokine gene expression in human skeletal muscle during concentric contraction: Evidence that IL-8, like IL-6, is influenced by glycogen availability. Am. J. Physiol. Regul. Integr. Comp. Physiol..

[35] Beavers K.M., Hsu F.C., Isom S., Kritchevsky S.B., Church T., Goodpaster B., Pahor M., Nicklas B.J. (2010). Long-term physical activity and inflammatory biomarkers in older adults. Med. Sci. Sports Exerc..

[36] Dyson S. (2021). The Ridden Horse Pain Ethogram. Equine Vet. Educ..

[37] Crass J.R., Genovense R.L., Render J.A., Bellon E.M. (1992). Magnetic resonance, ultrasound and histopathologic correlation of acute and healing tendon injuries. Vet. Radiol. Ultrasound.

[38] Dyson S., Murray R. (2012). Management of hindlimb proximal suspensory desmopathy by neurectomy of the deep branch of the lateral plantar nerve and plantar fasciotomy: 155 horses (2003–2008). Equine Vet. J..

[39] Docheva D., Müller S.A., Majewski M., Evans C.H. (2015). Biologics for tendon repair. Adv. Drug Deliv. Rev..

[40] Durgam S., Stewart M. (2017). Cellular and Molecular Factors Influencing Tendon Repair. Tissue Eng. Part B Rev..

[41] Scheller J., Chalaris A., Schmidt-Arras D., Rose-John S. (2011). The pro- and anti-inflammatory properties of the cytokine interleukin-6. Biochim. et Biophys. Acta.

[42] Dakin S.G., Werling D., Hibbert A., Abayasekara D.R., Young N.J., Smith R.K., Dudhia J. (2012). Macrophage sub-populations and the lipoxin A4 receptor implicate active inflammation during equine tendon repair. PLoS ONE.

[43] de Castro Pochini A., Ejnisman B., de Seixas Alves M.T., Uyeda L.F., Nouailhetas V.L., Han S.W., Cohen M., Albertoni W.M. (2011). Overuse of training increases mechanoreceptors in supraspinatus tendon of rats SHR. J. Orthop. Res. Off. Publ. Orthop. Res. Soc..

[44] Gilles C.L. (2016). Rehabilitation of tendon and ligament injuries. Proc. Am. Assoc. Equine Pract. Cong Orlando US.

[45] Lui P.P.Y., Yung P.S.H. (2021). Inflammatory mechanisms linking obesity and tendinopathy. J. Orthop. Transl..

[46] Zhou S., Maleitzke T., Geissler S., Hildebrandt A., Fleckenstein F.N., Niemann M., Fischer H., Perka C., Duda G.N., Winkler T. (2022). Source and hub of inflammation: The infrapatellar fat pad and its interactions with articular tissues during knee osteoarthritis. J. Orthop. Res..

[47] Shetty G.K., Economides P.A., Horton E.S., Mantzoros C.S., Veves A. (2004). Circulating adiponectin and resistin levels in relation to metabolic factors, inflammatory markers, and vascular reactivity in diabetic patients and subjects at risk for diabetes. Diabetes Care.

[48] Calabro P., Samudio I., Willerson J.T., Yeh E.T. (2004). Resistin promotes smooth muscle cell proliferation through activation of extracellular signal-regulated kinase 1/2 and phosphatidylinositol 3-kinase pathways. Circulation.

[49] Verma S., Li S.H., Wang C.H., Fedak P.W., Li R.K., Weisel R.D., Mickle D.A. (2003). Resistin promotes endothelial cell activation: Further evidence of adipokine-endothelial interaction. Circulation.

[50] Kougias P., Chai H., Lin P.H., Lumsden A.B., Yao Q., Chen C. (2005). Adipocyte-derived cytokine resistin causes endothelial dysfunction of porcine coronary arteries. J. Vasc. Surg..

[51] Cho Y., Lee S.E., Lee H.C., Hur J., Lee S., Youn S.W., Lee J., Lee H.J., Lee T.K., Park J. (2011). Adipokine resistin is a key player to modulate monocytes, endothelial cells, and smooth muscle cells, leading to progression of atherosclerosis in rabbit carotid artery. J. Am. Coll. Cardiol..

[52] Steppan C.M., Brown E.J., Wright C.M., Bhat S., Banerjee R.R., Dai C.Y., Enders G.H., Silberg D.G., Wen X., Wu G.D. (2001). A family of tissue-specific resistin-like molecules. Proc. Natl. Acad. Sci. USA.

[53] Fatouros I.G., Chatzinikolaou A., Tournis S., Nikolaidis M.G., Jamurtas A.Z., Douroudos I.I., Papassotiriou I., Thomakos P.M., Taxildaris K., Mastorakos G. (2009). Intensity of resistance exercise determines adipokine and resting energy expenditure responses in overweight elderly individuals. Diabetes Care.

[54] Van Pelt D.W., Guth L.M., Horowitz J.F. (2017). Aerobic exercise elevates markers of angiogenesis and macrophage IL-6 gene expression in the subcutaneous adipose tissue of overweight-to-obese adults. J. Appl. Physiol..

[55] Lavie C.J., Ozemek C., Carbone S., Katzmarzyk P.T., Blair S.N. (2019). Sedentary Behavior, Exercise, and Cardiovascular Health. Circ. Res..

[56] Ihalainen J.K., Schumann M., Eklund D., Hämäläinen M., Moilanen E., Paulsen G., Häkkinen K., Mero A.A. (2018). Combined aerobic and resistance training decreases inflammation markers in healthy men. Scand. J. Med. Sci. Sports.

[57] Nikiforov A., Kulikova V., Ziegler M. (2015). The human NAD metabolome: Functions, metabolism and compartmentalization. Crit. Rev. Biochem. Mol. Biol..

[58] Kim D.H., Lee H.G., Sp N., Kang D.Y., Jang K.J., Lee H.K., Cho B.W., Yang Y.M. (2021). Validation of exercise-response genes in skeletal muscle cells of Thoroughbred racing horses. Asian-Australas. J. Anim. Sci..

[59] Buchbinder R., van Tulder M., Öberg B., Costa L.M., Woolf A., Schoene M., Croft P. (2018). Lancet Low Back Pain Series Working Group. Low back pain: A call for action. Lancet.

[60] Risbud M.V., Shapiro I.M. (2014). Role of cytokines in intervertebral disc degeneration: Pain and disc content. Nat. Rev. Rheumatol..

[61] Ackermann P.W., Hart D.A. (2016). General Overview and Summary of Concepts Regarding Tendon Disease Topics Addressed Related to Metabolic Disorders. Adv. Exp. Med. Biol..

[62] Sui Y., Lee J.H., DiMicco M.A., Vanderploeg E.J., Blake S.M., Hung H.H., Plaas A.H., James I.E., Song X.Y., Lark M.W. (2009). Mechanical injury potentiates proteoglycan catabolism induced by interleukin-6 with soluble interleukin-6 receptor and tumor necrosis factor alpha in immature bovine and adult human articular cartilage. Arthritis Rheum..

[63] Rechardt M., Viikari-Juntura E., Shiri R. (2014). Adipokines as predictors of recovery from upper extremity soft tissue disorders. Rheumatology.

[64] Wang M., Wei J., Li H., Ouyang X., Sun X., Tang Y., Chen H., Wang B., Li X. (2018). Leptin Upregulates Peripheral CD4+CXCR5+ICOS+ T Cells via Increased IL-6 in Rheumatoid Arthritis Patients. J. Interferon Cytokine Res. Off. J. Int. Soc. Interferon Cytokine Res..

[65] Kędzierski W. (2016). Leptin fluctuations in trained horses, during a work season. J. Equine Vet. Sci..

[66] Filková M., Haluzík M., Gay S., Senolt L. (2009). The role of resistin as a regulator of inflammation: Implications for various human pathologies. Clin. Immunol..

[67] Reynolds A., Keen J.A., Fordham T., Morgan R.A. (2019). Adipose tissue dysfunction in obese horses with equine metabolic syndrome. Equine Vet. J..

[68] Carter R.A., Dugdale A.H.A. (2013). Assessment of body condition and bodyweight. Equine Appl. Clin. Nutr..

[69] Werpy N.M., Denoix J.M., McIlwraith C.W., Frisbie D.D. (2013). Comparison between standard ultrasonography, angle contrast ultrasonography, and magnetic resonance imaging characteristics of the normal equine proximal suspensory ligament. Vet. Radiol. Ultrasound.

[70] Giardullo L., Corrado A., Maruotti N., Cici D., Mansueto N., Cantatore F.P. (2021). Adipokine role in physiopathology of inflammatory and degenerative musculoskeletal diseases. Int. J. Immunopathol. Pharmacol..

